# FcRn Blockade as a Targeted Therapeutic Strategy in Antibody-Mediated Autoimmune Diseases: A Focus on Warm Autoimmune Hemolytic Anemia

**DOI:** 10.3390/antib14030065

**Published:** 2025-08-01

**Authors:** Michael Sandhu, Irina Murakhovskaya

**Affiliations:** Montefiore Medical Center, Albert Einstein College of Medicine, Bronx, NY 10461, USA; msandhu@montefiore.org

**Keywords:** FcRn inhibitor, FcRn blocker, warm autoimmune hemolytic anemia

## Abstract

Antibody-mediated autoimmune diseases are common, can involve any organ system, and pose a large burden for patients and healthcare systems. Most antibody-mediated diseases are mediated by IgG antibodies. Selective targeting of pathogenic antibodies is an attractive treatment option which has already proven to be effective in antibody-positive generalized myasthenia gravis, maternal-fetal alloimmune cytopenias, and immune thrombocytopenic purpura. Warm autoimmune hemolytic anemia (wAIHA) is an autoimmune disorder mediated by pathogenic antibodies mainly of the IgG class with no approved therapy. Current treatment includes non-specific immunosuppression with corticosteroids, rituximab, and other immunosuppressive agents. With most therapies, time to response can be delayed and transfusions may be needed. Neonatal Fc receptor (FcRN) therapies provide rapid and sustained reduction of pathogenic IgG levels providing potential for fast, effective therapy in antibody-mediated autoimmune diseases including warm autoimmune hemolytic anemia. This review focuses on the emerging role of FcRn inhibition in autoimmune hematologic diseases, and their therapeutic potential in wAIHA.

## 1. Introduction

Autoimmune diseases affect roughly 10% of people in high-income countries such as the UK, with higher prevalence in women (13.1%) than men (7.4%), according to a large study of 22 million individuals [1]. Incidence has risen over the past two decades, especially for celiac disease, Sjögren’s syndrome, and Graves’ disease. Globally, prevalence ranges from 4% to 10%, with variation by region and sociodemographic factors [1,2,3,4,5].

These diseases pose a significant healthcare and economic burden, contributing to high disability, reduced quality of life, and increased healthcare use. The rise in prevalence and associated disability-adjusted life years (DALYs) highlights the urgent need for targeted health policies and resource planning [6]. Historically, autoimmune diseases were thought to exist as distinct disorders; however, some autoimmune diseases share pathophysiologic mechanisms and may occur together [7].

Most clinically significant autoimmune diseases are driven by IgG autoantibodies. IgG autoantibodies are known for their long half-life and potent effector functions. IgG is the main isotype involved in the pathogenesis of both systemic and organ-specific autoimmune disorders, including systemic lupus erythematosus (SLE), rheumatoid arthritis (RA), generalized myasthenia gravis (gMG), Graves’ disease, pemphigus vulgaris, immune thrombocytopenic purpura (ITP), and autoimmune hemolytic anemia (AIHA). These IgG-mediated diseases represent the largest portion of autoimmune prevalence and contribute substantially to global healthcare burden [8,9,10].

In organ-specific autoimmune diseases, IgG autoantibodies typically cause direct damage to target tissues. In systemic conditions like systemic lupus erythematosus, they can also bind to various ubiquitous intracellular antigens and contribute to disease through immune complex formation. Antibody effector functions can be categorized as direct (mediated by the variable region, such as steric hindrance or signal modulation) and indirect (mediated by the constant region, involving mechanisms like complement activation or Fc receptor engagement) [11].

FcRn (neonatal Fc receptor) inhibition is an emerging approach in antibody-mediated diseases such as AIHA. FcRn prevents IgG degradation by mediating its recycling and bidirectional transcytosis, extending half-life and maintaining a high plasma level [11,12,13]. Blocking FcRn accelerates clearance of pathogenic and nonpathogenic IgG without affecting production of immunoglobulins [14]. Reducing IgG levels by blocking its recycling offers a promising therapeutic strategy.

FcRn inhibitors offer several advantages over current AIHA treatments: they are well-tolerated, have a rapid onset of action, minimize infection risk, and serve as effective steroid-sparing agents for patients who cannot tolerate long-term corticosteroid therapy. These agents enhance IgG degradation, lowering levels of pathogenic IgG and immune complexes, without affecting IgA, IgM, IgE, complement, plasma cells, B cells, or other components of the innate or adaptive immune system [14,15].

This review focuses on the emerging role of FcRn inhibition in autoimmune hematologic diseases, and their therapeutic potential in wAIHA.

## 2. Warm Autoimmune Hemolytic Anemia

Autoimmune hemolytic anemia is a rare disorder with an incidence of 1–3 per 10^5^/year and a prevalence of 17:100,000 characterized by premature red blood cell (RBC) destruction driven by autoantibodies, with or without complement involvement [16,17,18]. Warm AIHA (wAIHA) accounts for 70–80% of cases of AIHA and involves mainly IgG, and rarely IgA, or IgM antibodies that react at 37 °C [17]. It can be characterized as primary or secondary to associated conditions such as hematologic malignancies, autoimmune diseases (e.g., SLE, RA, antiphospholipid syndrome), immune dysregulation, infections, or certain therapies (e.g., checkpoint inhibitors, hematopoietic and solid organ transplants) [19,20,21].

Clinical presentation ranges from compensated anemia to life-threatening hemolysis with a mortality rate of approximately 11% [22]. Autoantibodies, with or without complement fragments, on the RBC surface are detected using a direct antiglobulin test (DAT), also known as a direct Coombs test, although 5–10% of patients with wAIHA may be DAT negative [23].

## 3. Current Warm Autoimmune Hemolytic Anemia Treatment

Historically, wAIHA management has relied on corticosteroids as first-line therapy, guided mainly by expert opinion and limited clinical trial evidence [16,17,24,25,26,27,28,29]. Prednisone induces a response in 70–85% of patients within 2–3 weeks; however, durable remission is achieved in only about one-third [1]. Gradual tapering is essential to minimize relapse, often requiring treatment for six months or longer [25]. High or intravenous doses may be needed in severe cases [24,30,31].

Rituximab, an anti-CD20 monoclonal antibody, is used as adjunct therapy in patients with severe disease and in steroid-refractory or relapsed cases [24]. Rituximab non-specifically depletes B-cells and leads to generalized immunosuppression [25]. Dosing regimens include body surface area based 375 mg/m^2^ weekly for four weeks, or a fixed-dose regimen of 1000 mg administered as 2 doses 2 weeks apart, or a fixed-dose low dose (100 mg weekly for 4 doses) [32,33,34,35]. Rituximab yields overall response rate of 83–87%, with 72% disease-free survival at one year and 56% at two years [36].

Sirolimus, particularly effective in autoimmune lymphoproliferative syndrome (ALPS)-associated wAIHA, has demonstrated response rates of 70–80% in relapsed/refractory cases with autoimmune cytopenias including warm autoimmune hemolytic anemia [37,38]. Other immunosuppressants, such as azathioprine, cyclosporine, and mycophenolate mofetil, are also used, although supported by limited prospective data [16]. Therapeutic options for the management of AIHA are summarized in Table 1, along with their mechanism of action and noted toxicities.

Splenectomy is an option for refractory or steroid-intolerant patients, achieving remission in about two-thirds, though with surgical risks and potential long-term infectious complications [39,40,41]. In addition, long-term remission data are lacking.

Current treatments are associated with relapse and toxicity, due to mechanisms of B-cell escape [42]. Consequently, novel therapies are under investigation, including Spleen tyrosine kinase (Syk) and Phosphatidylinositol 3-kinase (PI3K) inhibitors, FcRn antagonists, Bruton’s tyrosine kinase (BTK) inhibitors, BAFF/APRIL pathway inhibitors, CD38 antibodies, and mammalian target of rapamycin (mTOR) inhibitors [43].

## 4. Neonatal Fragment Crystallizable Receptor (FcRn)

**Figure 1:**
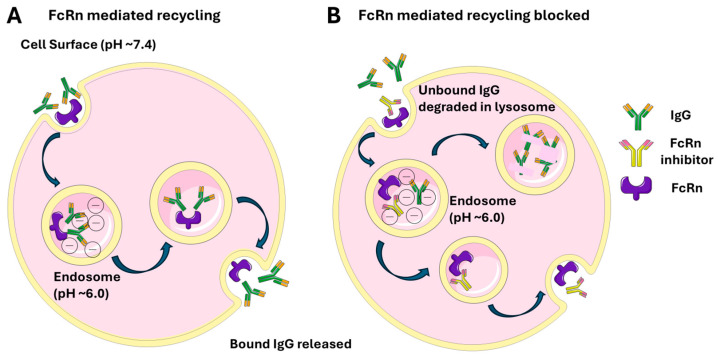
FcRn-mediated IgG recycling under physiologic conditions (**A**) and with FcRn inhibitor (**B**).

The neonatal Fc receptor (FcRn) is a heterodimer composed of β2-microglobulin and a membrane-bound α-chain structurally related to major histocompatibility complex (MHC) class I molecules [8,44,45]. Unlike classical MHC molecules, FcRn is non-polymorphic and not involved in classical antigen presentation. Initially recognized for mediating maternal IgG transfer across the placenta, FcRn expression persists beyond the neonatal period and is found in adult epithelial, endothelial, and hematopoietic cells. It plays a key role in immune regulation by extending IgG half-life through endosomal recycling and contributing to both innate and adaptive immune responses at both mucosal and systemic sites [13]. Neonatal Fc receptor (FcRn) mediates transport of antibodies across the cell membrane (Figure 1) by receptor-mediated transcytosis and is influenced by pH levels. There is high affinity at endosomal pH (approximately ~6.0) and low affinity at physiologic pH (~7.4).

The neonatal Fc receptor (FcRn) extends the half-life of IgG and albumin by protecting them from lysosomal degradation, unlike IgA and IgM, which lack such recycling. This recycling process returns IgG to the bloodstream, making IgG the most common antibody subtype in serum and is key to its long half-life of approximately 21 days [45,46] FcRn also mediates bidirectional IgG transcytosis, playing a role in immune surveillance at mucosal sites and antigen presentation via MHC I and II pathways modulating T cell responses [12,13].

A. Circulating IgG antibodies are internalized by endothelial or other cells via fluid-phase pinocytosis. Inside lysosomes (pH ~6.0), the acidic environment allows FcRn to bind the Fc region of IgG, preventing the antibodies from degradation. The FcRn-IgG complexes are trafficked back to the cell surface. Upon exposure to the neutral extracellular pH (~7.4), FcRn releases IgG, returning it to the bloodstream intact. B. In the presence of FcRn inhibitor unbound IgG is degraded in the acidic pH of lysosomes.

During pregnancy, FcRn is the primary placental transporter responsible for the transfer of maternal IgG to the fetus, using a similar pH-dependent transcytosis mechanism to move IgG across the placental barrier [47,48].

FcRn blockade selectively lowers pathogenic IgG without impacting other immunoglobulins, a mechanism validated in clinical trials for autoimmune diseases like myasthenia gravis and ITP [14,15,49]. FcRn-targeting therapies under development include nipocalimab, batoclimab, efgartigimod and IMVT-1402, and IgG4 FcRn targeting antibodies rozanolixizumab and STSA-1301. Of these, nipocalimab is the only FcRn-targeted agent currently in clinical trials for warm autoimmune hemolytic anemia (wAIHA). A review of FcRn inhibitors approved and in late clinical development and key clinical trials is shown in Table 2.

## 5. Nipocalimab

Nipocalimab is a fully human, aglycosylated, effectorless immunoglobulin G1 (IgG1) monoclonal antibody engineered to lack Fc effector functions, including C1q and activating Fcγ receptor binding, that selectively binds with high affinity to the IgG-binding site on FcRn, blocking IgG recycling [15,50,51]. In cynomolgus monkeys, nipocalimab induced dose-dependent IgG reductions of ~50–70% by weeks 4–8, returning to baseline eight weeks after the final dose. Immune responses to T-dependent neoantigens remained intact, with no treatment-related changes in peripheral blood lymphocyte counts or subsets, cytotoxic T-cell, NK, monocyte, or granulocyte function or cytokine levels [51].

In clinical studies, nipocalimab selectively reduced total IgG (all subclasses) by up to ~85%, sustained ≥75% reductions for 24 days, without affecting IgM, IgA, IgE, complement activity (CH50, C3, C4), inflammatory cytokines, or acute phase proteins such as C-reactive protein [52]. Importantly, it does not interfere with the new antibody production [21].

In the phase 2a IRIS-RA study—a randomized, double-blind, placebo-controlled trial in patients with moderate to severe rheumatoid arthritis—nipocalimab-treated patients maintained pre-existing anti-tetanus and anti-varicella IgG levels above protective threshold and were able to mount IgG responses to SARS-CoV-2 infection, developing antibodies to both spike (S1 RBD) and nucleocapsid proteins. Four patients contracted COVID-19 during treatment; three cases were mild and one moderate all resolved without complications [53].

The impact of nipocalimab on vaccine-induced IgG responses was assessed in a phase 1 open-label, randomized study in healthy adults receiving T-cell-dependent and independent vaccines (i.e., tetanus toxoid (TT), diphtheria, and acellular pertussis vaccine (Tdap), and 23-polysaccharide pneumococcal vaccine (PPSV^®^2), respectively). At Week 4, the median reduction in total IgG was 65.9% in the nipocalimab group, compared to an 8.2% increase in controls, returning to baseline by Week 16. All participants mounted a Tdap response, with 20% vs. 50% achieving positive anti-TT responses (*p* = 0.089). While anti-TT and anti-pneumococcal IgG levels were lower in the nipocalimab group at Week 4, responses were comparable by Week 16, and protective antibody thresholds were maintained in all participants. Nipocalimab was well tolerated, supporting the feasibility of continued adherence to routine vaccination schedules during treatment [54].

Nipocalimab is currently studied in the ENERGY trial, where participants ≥ 18 years of age who had been diagnosed with wAIHA for at least 3 months and are currently on treatment or had previously received treatment were randomized to either placebo or nipocalimab (at 2 dose levels) during the double-blind period and subsequently nipocalimab during the open-label extension [50,55]. The primary endpoint in this study is durable response in hemoglobin improvement, defined as hemoglobin >10 g/dL and increase from baseline in hemoglobin of >2 g/dL at 3 consecutive visits at or before week 16 without the need for rescue therapy. The study has been completed but results have not been released.

### 5.1. Nipocalimab in Other Indications

Nipocalimab has been investigated in other autoimmune diseases. Nipocalimab is currently approved for treating generalized myasthenia gravis (gMG) in adults and adolescents aged 12 years and older who test positive for anti-acetylcholine receptor (AChR) or anti-muscle-specific tyrosine kinase (MuSK) antibodies [56] and is under investigation for other IgG-driven conditions, such as hemolytic disease of the fetus and newborn [57], Sjogren Syndrome [58], chronic inflammatory demyelinating polyneuropathy [59], Fetal and Neonatal Alloimmune Thrombocytopenia [60], Systemic Lupus Erythematosus [61] and lupus nephritis [62] and rheumatoid arthritis.

#### 5.1.1. Myasthenia Gravis

Myasthenia gravis is an autoimmune disease marked by fluctuating muscle weakness due to impaired neuromuscular transmission. It is caused by autoantibodies—most commonly against acetylcholine receptor (AChR), but also against muscle-specific kinase (MuSK) or lipoprotein receptor–related protein 4 (LRP4)—that disrupt postsynaptic signaling, leading to reduced receptor function and fatigable weakness [63]. Similarly to wAIHA, conventional therapeutic agents used in the treatment of myasthenia gravis demonstrate a significant side effect profile and long latency for efficacy. Phase 2 data across multiple nipocalimab dose levels showed dose-dependent improvements in MG-ADL scores with no associated safety concerns [64].

The VIVACITY-MG3 trial, a pivotal phase 3 randomized, double-blind, placebo-controlled study, assessed the safety and efficacy of nipocalimab in adults with gMG. Conducted across 81 sites in 17 countries, the trial enrolled 199 patients who were positive for AChR, MuSK or LRP4 antibodies. Nipocalimab treatment resulted in dose-dependent reductions in total IgG and pathogenic autoantibodies, accompanied by meaningful improvements on the Myasthenia Gravis Activities of Daily Living (MG-ADL) scale [65]. Results from the open-label extension phase demonstrated sustained, clinically meaningful disease control over 84 weeks in a broad pop, isolation of autoantibody-positive gMG patients, with a consistent safety profile. Among participants receiving steroids at baseline, 45% (40/89) were able to reduce or discontinue steroid use, with the average prednisone dose decreasing from 23 mg to 10 mg/day while maintaining clinical efficacy. No new safety signals emerged, and adverse event rates remained consistent with those observed in the double-blind phase, despite ongoing IgG reduction [66], which often have delayed efficacy and notable side effects. Overall, the trial suggests nipocalimab may offer safe, effective, well-tolerated and sustained disease control with a relatively rapid onset compared to conventional therapies in a broad spectrum of antibody-positive gMG patients [65,67].

#### 5.1.2. Sjögren’s Disease

Sjögren’s disease (SjD) is a chronic autoimmune disease characterized by lymphocytic infiltration and dysfunction of the exocrine glands, most notably the salivary and lacrimal glands, resulting in the hallmark symptoms of dry mouth (xerostomia) and dry eyes (keratoconjunctivitis sicca). It is caused by dysregulated B-cell activity and elevated IgG autoantibodies, particularly anti-Ro and anti-La, which are linked to disease severity and represent therapeutic targets in the management of the disease [68].

The phase 2 DAHLIAS study (NCT04968912) was a multicenter randomized, double-blind, placebo-controlled trial which evaluated intravenous nipocalimab in adults with moderate-to-severe, anti-Ro antibody–positive primary Sjögren’s disease in addition to standard of care. Patients received 5 or 15 mg/kg nipocalimab or placebo every two weeks. At Week 24, the 15 mg/kg dose significantly improved disease activity measured by total Clinical European League Against Rheumatism Sjögren’s Syndrome Disease Activity Index (clinESSDAI score) versus placebo (LS mean difference −2.65; *p* = 0.002), with consistent benefit across secondary endpoints. The 5 mg/kg dose showed no significant effect. Nipocalimab was well tolerated, with similar rates of serious adverse events across groups and no treatment-related severe infections. These results provide proof of concept for FcRn blockade in SjD and support further development of nipocalimab [69]. Nipocalimab, is the first investigational therapy to receive FDA Breakthrough Therapy designation for moderate-to-severe Sjögren’s disease, and has been granted Fast Track status [70].

Phase 3 DAFFODIL study is a randomized, double-blind, placebo-controlled study currently evaluating the efficacy, safety, and tolerability of nipocalimab in adults (≥18 years) with moderate-to-severe Sjögren’s disease [58].

#### 5.1.3. Hemolytic Disease of the Fetus and Newborn

Hemolytic disease of the fetus and newborn (HDFN) is an alloimmune disorder where maternal IgG antibodies target paternally inherited fetal RBC antigens. These antibodies cross the placenta, leading to fetal RBC destruction and anemia, which can be life-threatening and can lead to hydrops fetalis, heart failure, or death if untreated [71]. While RhD is the most common trigger, other antigens like Kell, Duffy, Kidd, and additional Rh antigens (C, c, E, e) have also been implicated [72]. Intrauterine transfusions (IUT) are the mainstay of therapy, but are invasive, technically complex, and limited to specialized centers [73,74]. Risks, including preterm premature rupture of membranes (pPROM) and fetal death, are higher when performed before 24 weeks of gestation [74,75]. Early-onset severe HDFN—defined as occurring at or before 24 weeks’ gestation—is linked to significant fetal and neonatal morbidity and mortality.

Blocking FcRn, the sole placental IgG transporter and salvage receptor that maintains circulating maternal serum IgG levels, is intended to reduce maternal alloantibody titers and limit transplacental IgG transfer to the fetus. The UNITY study (NCT03842189), was an international proof-of-concept, open-label phase 2 trial evaluating intravenous nipocalimab (30 or 45 mg/kg/week) beginning between 14 and 35 weeks’ gestation in 13 pregnancies at high risk for recurrent early-onset severe HDFN [76]. Nipocalimab treatment delayed or prevented fetal anemia and intrauterine transfusions in high-risk pregnancies, without additional serious adverse events. Overall, 12 of 13 pregnancies (92%) resulted in live births, with 7 (54%) requiring no intrauterine transfusions and a median delivery at 36 weeks and 4 days. Notably, 6 participants (46%) needed no antenatal or neonatal transfusions. A Phase 3 Randomized, Placebo-Controlled, Double-Blind, Multicenter Study to Evaluate the Efficacy and Safety of Nipocalimab in Pregnancies at Risk for Severe Hemolytic Disease of the Fetus and Newborn (HDFN) is currently recruiting patients [57].

## 6. Other FcRn Targeting Agents

### 6.1. Efgartigimod

Efgartigimod is a human IgG1 antibody Fc fragment engineered to enhance FcRn affinity while preserving its pH-dependent interaction profile that blocks FcRn [77] with a long clinical plasma half-life. Efgartigimod is unique among FcRn antagonists in that it lacks engineered FcRn-specific variable regions and instead mimics the natural Fc:FcRn interaction, rather than blocking FcRn via Fab binding. It has been studied in patients with autoimmune disease mediated by pathogenic IgG autoantibodies. In a study of 62 healthy volunteers (NCT03457649), efgartigimod induced rapid, dose-dependent reductions of up to 50% in total IgG and all IgG subtypes after a single dose, with levels returning to baseline within approximately 8 weeks [49].

In the phase 3, double-blind, placebo-controlled ADAPT trial, efgartigimod (10 mg/kg) administered in cycles of 4-weekly infusions, repeated as needed after 8 weeks based on response, was well tolerated and led to improved symptom control in antibody-positive gMG [78]. A post hoc analysis of the ADAPT study showed that efgartigimod maintained its effectiveness across multiple treatment cycles, supporting its potential as a long-term therapy [79].

In immune thrombocytopenia (ITP) IgG autoantibodies can accelerate platelet clearance, impair platelet production, trigger platelet apoptosis or complement-mediated lysis, and potentially disrupt platelet function [80]. In a phase 2 study of 38 heavily pretreated adult patients with ITP randomized to receive efgartigimod 5 or 10 mg/kg IV weekly for 4 weeks or placebo, efgartigimod rapidly reduced total IgG, increased platelet counts, and lowered bleeding rates compared to placebo [81].

In ADVANCE IV, a phase 3 multicenter, randomized, placebo-controlled trial efgartigimod administered intravenously at 10 mg/kg weekly for four weeks, with subsequent dosing intervals individualized based on platelet response, significantly increased sustained platelet count responses compared with placebo in adults with chronic ITP who had failed multiple prior therapies [82]. The primary endpoint of sustained platelet count response (≥50 × 10^9^ for at least 4 of the last 6 weeks) was reached in 22% of patients receiving efgartigimod compared with 5% of those receiving placebo (*p* = 0.032). Efgartigimod was well tolerated, with most adverse events mild or moderate, and rates of infection and serious adverse events comparable to placebo.

The subcutaneous formulation of efgartigimod, combined with recombinant human hyaluronidase PH20 (VYVGART Hytrulo), was shown to be effective and non-inferior to intravenous administration in patients with generalized myasthenia gravis (gMG) and is approved for this indication [83,84]. It is also approved for chronic inflammatory demyelinating polyradiculoneuropathy (CIDP) based on results from the ADHERE trial [85].

However, in the Phase 3 ADVANCE-SC trial for ITP, the primary endpoint of sustained platelet response and key secondary endpoints were not met, despite significant IgG reduction—underscoring the complex and variable link between IgG lowering and clinical response in ITP [86].

### 6.2. Batoclimab

Batoclimab is a fully humanized IgG1 monoclonal antibody targeting the neonatal Fc receptor (FcRn) [87]. In healthy volunteers, a single 680 mg subcutaneous dose reduced serum IgG levels by about 40% by day 11, and four weekly doses achieved up to a 75% reduction in total IgG [88]. In a multicenter, randomized, placebo-controlled trial conducted at 27 centers in China, 132 adults with antibody-positive myasthenia gravis received batoclimab or placebo in 6-week cycles of 680 mg subcutaneous injections, followed by a 4-week observation period. Batoclimab was well tolerated and resulted in a significantly higher rate of sustained MG-ADL symptom improvement compared to placebo (58% vs. 31%) [89].

Batoclimab is currently being studied in other IgG-mediated autoimmune disorders, including thyroid eye disease, and neuromyelitis optica spectrum disorder. IgG autoantibodies directed against the TSH receptor (TSH-R) on thyroid and orbital cells drive the pathogenesis of Graves’ disease and its associated manifestation, thyroid eye disease (TED). Inhibition of FcRn reduces pathogenic thyrotropin receptor antibodies (TSH-R-Ab). A proof-of-concept trial demonstrated marked decreases in anti-TSH-R-Ab and total IgG serum levels with batoclimab [90]. However, batoclimab did not significantly improve proptosis at 12 weeks vs. placebo, though earlier timepoints showed benefit. Orbital muscle volume decreased by week 12 (*p* < 0.03), and quality of life (appearance score) improved by week 19 in the 680 mg group (*p* < 0.03). The trial was terminated early because of unanticipated increases in serum cholesterol levels, which were reversed upon discontinuation of Batoclimab.

Neuromyelitis optica spectrum disorder (NMOSD) is a severe autoimmune neurological inflammatory disease driven mainly by pathogenic aquaporin-4 antibodies (AQP4-IgG antibodies). In a phase 1b open-label, dose-escalation study, nine patients with acute myelitis or optic neuritis received four weekly subcutaneous doses of batoclimab (340 mg or 680 mg) alongside standard intravenous methylprednisolone pulse. Batoclimab was well tolerated with no serious adverse events. In the 680 mg group, IgG levels reached maximum reduction by day 22, and AQP4-IgG became undetectable in six of seven patients. The Expanded Disability Status Scale score improved by 1.3 points at week 4 compared to baseline [91].

### 6.3. IMVT-1402

IMVT-1402 is a next-generation anti-FcRn monoclonal antibody designed to bind to the FcRn receptor and prevent IgG recycling which has been studied in both healthy patients and patients with autoimmune diseases [92]. In Phase 1 single ascending dose (SAD) and 300 mg multiple ascending dose (MAD) studies in healthy adults IMVT-1402 demonstrated dose-dependent reductions in IgG levels, comparable to those achieved with high-dose batoclimab (~60–74%), without significant effects on albumin or LDL-C. In the 600 mg MAD cohort, four weekly subcutaneous doses resulted in a mean IgG reduction of 74%, again with minimal impact on albumin and lipid levels [93].

### 6.4. Rozanolixizumab

Rozanolixizumab is a humanized IgG4 kappa anti-FcRn monoclonal antibody targeting the IgG-binding region of FcRn currently FDA-approved for the treatment of antibody-positive generalized myasthenia gravis. IgG4 antibodies have limited capacity to engage immune effector molecules compared to IgG1 subclass. Rozanolixizumab binds human FcRn with picomolar affinity over 600 times stronger than the affinity reported for efgartigimod at pH 6.0 in independent studies and demonstrates minimal pH dependence. It is administered as a weight-based subcutaneous infusion weekly for 6 weeks, with further dosing guided by clinical response [94]. In preclinical and early clinical trials it reduced serum IgG concentration without affecting IgA, IgM, or IgE levels or albumin levels [95]. In a randomized, placebo-controlled phase 3 MycarinG trial, it significantly reduced total and pathogenic IgG levels, leading to symptom improvement in patients with antibody-positive generalized myasthenia gravis [96].

Rozanolixizumab was also studied in a phase 2, multicenter multiple-dose study in 66 patients with persistent/chronic primary ITP [97]. Rozanolixizumab was well tolerated, with clinically meaningful platelet responses (≥50 × 10^9^/L) achieved in 66.7% and 54.5% of patients receiving single doses of 15 and 20 mg/kg, respectively, with responses observed as early as day 8. Efficacy correlated with rapid IgG reductions, while other immunoglobulin and albumin levels remained stable [97]. Two prematurely terminated 24-week randomized, double-blind, placebo-controlled phase 3 studies (TP0003, TP0006) and their 52-week open-label extension (OLE) evaluated efficacy of rozanolixizumab in 63 adults with persistent/chronic ITP [98]. Due to early termination, small numbers were randomized to rozanolixizumab or placebo (TP0003: 21 vs. 12; TP0006: 20 vs. 10), the sample size was too small to allow for statistically meaningful comparisons of primary and secondary endpoints between groups. Durable clinically meaningful platelet response (DCMPR), platelets ≥50 × 10^9^/L for ≥8 of 12 weeks, was observed only in rozanolixizumab-treated patients (TP0003: 4/21; TP0006: 1/20). By Day 8, platelet counts rose to ≥50 × 10^9^/L in 52.4% (TP0003) and 45.0% (TP0006) of treated patients. In the OLE, 21/43 patients who were switched to weekly dosing maintained platelet responses, but not with biweekly dosing. This is potentially related to interference with pH independent FcRn-mediated recycling caused by rozanolixizumab binding to FcRn, leading to rozanolixizumab accelerated clearance [96,99]. Adverse events were consistent with prior data, most commonly headache, pyrexia, and nausea [98].

### 6.5. STSA-1301

STSA-1301 is a recombinant anti-human FcRn humanized IgG4 monoclonal antibody [100], that functions in a pH-independent manner to bind FcRn and prevent IgG recycling. Efficacy of STSA-1301 has been demonstrated in mouse models, and it is currently being studied in phase 1/2 clinical trials for ITP [101,102].

## 7. Discussion

Warm autoimmune hemolytic anemia (wAIHA) is a rare condition without approved therapies. While many patients initially respond to corticosteroids, relapses are frequent, and second-line options like rituximab carry infection risks and may not effectively target long-lived plasma cells—the main source of autoantibodies. Third-line therapies often rely on broad immunosuppression with added toxicity.

FcRn blockade offers a targeted alternative by accelerating degradation of IgG, including pathogenic autoantibodies, through disruption of the IgG recycling pathway. Unlike conventional immunosuppressants, FcRn inhibitors lower IgG selectively without affecting IgA, IgM, or immune cells, and they preserve vaccine responses, reducing the risk of infections and malignancy.

FcRn inhibitors demonstrate a rapid onset of action, producing dose-dependent reductions in total IgG levels within days, which frequently correlates with early clinical improvement, making FcRn inhibition a targeted, less invasive alternative to plasma exchange (PLEX) in the treatment of IgG-mediated autoimmune diseases. PLEX removes circulating IgG antibodies non-specifically, whereas FcRn inhibitors allow for a more sustained and selective reduction in IgG without removing other plasma components. Clinical trials of FcRn inhibitors, such as efgartigimod and rozanolixizumab, have demonstrated comparable reductions in pathogenic IgG levels to those achieved with PLEX in diseases like myasthenia gravis and immune thrombocytopenia, with fewer procedure-related risks and greater patient convenience [80,83,85]. As FcRn inhibitors continue to show efficacy in clinical trials, they may emerge as a preferable alternative to PLEX in many settings, particularly in outpatient management and in patients with contraindications to invasive therapies.

Structural differences among anti-FcRn therapies contribute to their varying efficacy, safety, and pharmacokinetics. Efgartigimod, an IgG1 Fc fragment lacking engineered Fab regions, mimics natural Fc:FcRn interactions, unlike full-length antibodies such as batoclimab and nipocalimab, which inhibit FcRn via antigen-Fab binding. These structural distinctions affect FcRn trafficking: efgartigimod increases FcRn expression, while other agents promote its degradation or retention [103]. Additionally, full-length antibodies can bind more FcRn molecules due to their bivalent Fab and Fc domains. The lack of pH-dependent dissociation also limits recycling of FcRn blockers, contributing to non-linear pharmacokinetics seen with agents like efgartigimod and rozanolixizumab [94].

Another distinguishing effect among FcRn antagonists is their impact on albumin levels, a known adverse effect of some agents in this class. In the head-to-head study, repeated dosing with a recombinant analog of efgartigimod led to transient increases of up to 10% in circulating albumin in transgenic mice expressing human albumin and FcRn. In contrast, recombinant analogs of batoclimab and nipocalimab were associated with varying degrees of albumin reduction [103].

Some studies evaluating FcRn-blocking therapies have shown limited or inconsistent clinical benefit. For instance, in pemphigus, efgartigimod did not lead to a significantly higher rate of complete remission compared to placebo [104]. In ITP, intravenous efgartigimod demonstrated improvements in platelet counts and reductions in IgG levels in the Phase 3 ADVANCE IV trial [85]. However, these results were not replicated in the subsequent ADVANCE-SC trial, which assessed a subcutaneous formulation co-formulated with hyaluronidase (VYVGART Hytrulo^®^) in 207 patients with chronic or persistent ITP [89]. Similarly, in two Phase 3 ITP studies of rozanolixizumab, although every-2-week dosing led to sustained IgG reduction, platelet count increases were not consistently maintained throughout the dosing interval. Greater IgG reductions were observed with weekly dosing, but still did not consistently translate into durable platelet responses [98]. These findings suggest that reducing circulating IgG alone may not be sufficient for clinical improvement in all IgG-mediated diseases, likely due to disease heterogeneity and contributions from other immune mechanisms beyond pathogenic IgG autoantibodies.

An important consideration is the potential role of FcRn blockade in patients with multiple coexisting IgG-mediated autoimmune diseases. Although this application has not been directly studied, the mechanism of action—targeting pathogenic IgG autoantibodies—may offer particular benefit in this population. Patients with polyautoimmunity often require overlapping or sequential therapies for each condition. FcRn inhibitors, by addressing a shared immunopathogenic pathway, could suppress disease activity across multiple IgG-mediated conditions simultaneously. This approach may reduce the need for multiple immunosuppressive agents, thereby minimizing polypharmacy, lowering treatment-related toxicity (including immunosuppression), and improving treatment adherence.

Additionally, patients with autoimmune disease flare-ups may often require additional immunosuppression including corticosteroids. In patients receiving concurrent immunosuppression alongside FcRn inhibition, there may be an increased risk for infection and these patients should be closely monitored and counseled on the potential risks. However, due to non-IgG antibody class sparing, serious infection has not been a significant concern and FcRn inhibitors have demonstrated favorable safety profiles [105]. In a systematic review published in early 2024 assessing the safety and efficacy of FcRn inhibitors in patients with myasthenia gravis with the exception of rozanolixizumab all FcRn inhibitors demonstrated safety profiles comparable to placebo [106].

As FcRn inhibitors become more available as a treatment option in various disease states and move towards broader application, future challenges may arise in both transitioning to an FcRn inhibitor from prior therapy as well as potential long-term consequences of their use [107]. In the phase II AHERE study, patients with CIDP were monitored when taken off prior therapy prior to starting efgartigimod [85]. However, in real-world data from Levine at al., nine patients were transitioned from IVIG to efgartigimod with four experiencing severe relapse of their CIDP [107]. This raises the concern of potential disease flare or relapse when switching therapies.

Furthermore, due to short follow-up in clinical trials, the long-term effects of FcRn inhibitors are not well established. In specific, data regarding drug interactions with other immunomodulatory medications and the timing and efficacy of vaccinations in these patients will be helpful to better understand long-term management [108]. Cost of therapy, when compared to the current treatment landscape, may also be a limitation to the use of FcRn inhibitors but have to be weighed against cost of other healthcare resource utilization. Commonly used agents in autoimmune diseases including corticosteroids, rituximab, cyclosporine and sirolimus have been widely available and may be a more cost-effective option, however FcRn inhibitors offer a fast acting, safe steroid-sparing approach and have a role in acute disease management as well as after failure of prior therapies.

As these drugs become more readily available, future treatment considerations will include identifying markers that may predict response to FcRn inhibitors. The INFORM trial is currently ongoing to identify clinical, biological, cellular and genetic markers that make predict favorable response to FcRn inhibitors in patients with myasthenia gravis [109].

## 8. Conclusions

FcRn blockade offers a novel, targeted, and non-immunosuppressive approach to managing IgG-driven autoimmune diseases by lowering pathogenic antibody levels, improving symptoms, and potentially reducing reliance on corticosteroids and other broad immunosuppressants.

While phase III clinical trial data in various autoimmune disease settings, including wAIHA, MG, RA, Sjögren’s disease, and ITP have shown promising results, these studies are limited by small sample size and short follow-up periods. Open-label extensions of prior phase III clinical trials and real-world evidence data will help provide further information on the long-term efficacy and safety of FcRn inhibitors and the sustainability of the response. Furthermore, longer follow-up and additional real-world data will be helpful to further our understanding, especially in diverse patient populations and patients with other comorbid conditions including polyautoimmunity.

Challenges that may arise as FcRn inhibitors become more available and widely established include how to best transition from prior therapy to avoid disease flares, the potential long-term adverse effects, and identifying biomarkers that may predict which patients will respond. Additionally, real-world data on diverse patient populations and long-term follow-up are needed to improve our understanding of FcRn inhibitors.

## Figures and Tables

**Table 1:** Currently used therapies in warm autoimmune hemolytic anemia.

Therapy	Response Rate	Time to Response	Toxicities	Comments
Corticosteroids [16,24,26,27]	70–80%	2–3 weeks	Weight gain Hyperglycemia Peptic ulcer Adrenal insufficiency Delirium Myopathy Osteoporosis	To reduce risk of relapse, patients require long-term slow taper of steroids after reaching hemoglobin goal
Rituximab [33,34,35]	80%	3–6 weeks	Hypogammaglobulinemia Infection Impaired vaccine response	Can be used first-line in severe cases or second line for patients with steroid-refractory disease
Azathioprine [24,25,26,27]	60%	1–3 months	Increased infection risk Hepatotoxicity	
Cyclosporine [24,25,26]	60%	1–3 months	Increased infection risk Nephrotoxicity	
Cyclophosphamide [16,24,25,26]	50–70%	1–2 months	Increased infection risk Teratogen	
Splenectomy [39,40]	80%	1–2 weeks	Increased infection risk with encapsulated organism Thrombosis risk	Long-term efficacy unknown
Sirolimus [37]	79.5%	2 months	Increased infection risk Hepatotoxicity Mucositis	Particularly effective in autoimmune lymphoproliferative syndrome

**Table 2:** FcRn inhibitors approved and in late clinical development.

Therapy	Disease Indication	Dosing Regimen	Toxicities	Trial Phases
Nipocalimab	wAIHA RA gMG HDFN Sjögren’s FNAIT	IV Every 2 weeks	Increased risk of infection Infusion reactions	Phase II/III—NCT04119050 Phase II—NCT04991753 Phase II—NCT04968912 Phase II—NCT03842189 Phase III—NCT06741969 Phase III—NCT04951622
Efgartigimod	gMG ITP CIDP	IV, SQ Weekly or every 4–8 Weeks	Increased risk of infection Arthralgias Myalgias Injection-site reactions	Phase III—NCT03669588 Phase III—NCT04687072 Phase III—NCT04281472
Batoclimab	NMOSD gMG Thyroid eye disease	SC weekly × 6	Injection-site reactions Hypoalbuminemia Hypogammaglobulinemia Headache Hypercholesterolemia	Phase III—NCT05039190 Phase III—NCT05403541
Rozanolixizumab	gMG ITP	SC weekly	Increased infection risk Headache Pyrexia Nausea	Phase III—NCT03971422 Phase II—NCT03052751 Phase I—NCT02220153

CIDP chronic inflammatory demyelinating polyradiculoneuropathy, HDFN—Hemolytic Disease of the Fetus and Newborn, FNAIT Fetal and Neonatal Alloimmune Thrombocytopenia, ITP—immune thrombocytopenic purpura, IV—intravenous, gMG—generalized myasthenia gravis, NMSOD—neuromyelitis optica spectrum disorder, SC—subcutaneous, wAIHA—Warm Autoimmune Hemolytic Anemia.
